# Establishment of a Genome Editing Tool Using CRISPR-Cas9 in *Chlorella vulgaris* UTEX395

**DOI:** 10.3390/ijms22020480

**Published:** 2021-01-06

**Authors:** Jongrae Kim, Kwang Suk Chang, Sangmuk Lee, EonSeon Jin

**Affiliations:** Department of Life Science, Research Institute for Natural Sciences, Hanyang University, Seoul 04763, Korea; kjr1210@hanmail.net (J.K.); kschang@hanyang.ac.kr (K.S.C.); warmmuk@naver.com (S.L.)

**Keywords:** *Chlorella vulgaris*, genome editing, CRISPR-CAS9, nitrate reductase, adenine phosphoribosyltransferase, auxotrophic strain

## Abstract

To date, *Chlorella vulgaris* is the most used species of microalgae in the food and feed additive industries, and also considered as a feasible cell factory for bioproducts. However, the lack of an efficient genetic engineering tool makes it difficult to improve the physiological characteristics of this species. Therefore, the development of new strategic approaches such as genome editing is trying to overcome this hurdle in many research groups. In this study, the possibility of editing the genome of *C. vulgaris* UTEX395 using clustered regularly interspaced short palindromic repeats (CRISPR)-associated protein 9 (Cas9) has been proven to target nitrate reductase (*NR*) and adenine phosphoribosyltransferase (*APT*). Genome-edited mutants, *nr* and *apt*, were generated by a DNA-mediated and/or ribonucleoprotein (RNP)-mediated CRISPR-Cas9 system, and isolated based on the negative selection against potassium chlorate or 2-fluoroadenine in place of antibiotics. The null mutation of edited genes was demonstrated by the expression level of the correspondent proteins or the mutation of transcripts, and through growth analysis under specific nutrient conditions. In conclusion, this study offers relevant empirical evidence of the possibility of genome editing in *C. vulgaris* UTEX395 by CRISPR-Cas9 and the practical methods. Additionally, among the generated mutants, *nr* can provide an easier screening strategy during DNA transformation than the use of antibiotics owing to their auxotrophic characteristics. These results will be a cornerstone for further advancement of the genetics of *C. vulgaris*.

## 1. Introduction

Microalgae are photoautotrophic eukaryotic organisms that have potential as producers of biofuel and biomaterials, and significantly contribute to ecological processes by reducing atmospheric CO_2_ concentrations [1,2,3]. Among microalgal species, those belonging to the *Chlorella* genus, the most widely used microalgae in industry, are considered promising hosts for the expression of therapeutic proteins or the production of high-value compounds [4]. Moreover, *Chlorella* spp. are advantageous because they grow fast and are easy to culture in large scale, both indoors and outdoors [5].

Despite the advantages of *Chlorella* spp., the instability of protein expression and the relatively low production yield of high-value chemicals impede the expansion of its commercial use [4,6]. To increase the industrial value of *Chlorella* spp., many researchers have been working on genetic engineering tools to optimize *Chlorella* spp. transformation, including the development of novel transformation methods and improved vector construction using appropriate promoters, enhancers, marker genes, and codon optimizations. Electroporation and bombardment are mainly used to transfer genes into cells, and antibiotics such as hygromycin and zeocin are used for the selection of transformants [7,8,9,10,11,12,13,14,15,16]. However, despite a decade of study, the development of toolkits for the overexpression of genes has been insufficient. Besides, although various studies have been reported, the genetic engineering of *Chlorella* spp. still suffers from low reproducibility and low efficiency [17].

Recently, a methodology for more accurate genetic modification has emerged as an alternative to the traditional transformation method mediated by DNA vectors. Such new emerging technology for specific gene knockout, called gene editing, is used to create specific double-stranded DNA cleavages [18]. Gene editing techniques using zinc-finger nuclease (ZFN), transcription activator-like effector nuclease (TALEN), or clustered regularly interspaced short palindromic repeats (CRISPR)-associated protein 9 (CRISPR-Cas9), allow to edit (cleave and knockout) specific locations on the genome more easily [18,19,20,21,22]. Among these, the CRISPR-Cas9 technique is the most actively developed in microalgae owing to the complexity and difficulty of the experimental design of ZFN and TALEN [23,24]. The CRISPR-Cas9 system requires three basic components: the nuclease Cas9 that cleaves dsDNA at a site three nucleotides away from the protospacer adjacent motif (PAM), a CRISPR RNA consisting of a complementary 20-nucleotide sequence to the target DNA (crRNA), and a trans-activating crRNA (tracrRNA). crRNA and tracrRNA can, having physically fused, form a single guide RNA (sgRNA) [25]. In microalgae, the CRISPR-Cas9 system has been applied since 2014, initially with a DNA vector system [26,27] and recently using the ribonucleoprotein (RNP) system [28,29,30,31].

In this study, a genome editing technique mediated by CRISPR-Cas9 was tested to increase the potential of *C. vulgaris* UTEX395 as a model strain through genetic modification. Previously, phenotypic changes such as visual changes were used to improve the selection efficiency, however, these methods are less efficient and require considerable labor [20,30,32]. Therefore, targeting counter-selective markers such as *APT*, *NR*, peptidylprolyl isomerase (*FKB12*), tryptophan synthase beta subunit (*TSB*), or orotidine 5ʹ-phosphate decarboxylase (*UMP*) by genome editing has recently been proposed [29,31,33,34,35]. This strategy has the advantage of minimizing the insertion of foreign genes, unlike the recently reported method using the antibiotic resistance gene in *C. vulgaris* [36]. Accordingly, we targeted *NR* and *APT* to evaluate the possibility of genome editing by CRISPR-Cas9 in *C. vulgaris*. Besides, we applied RNP-based methodology including proteolistic bombardment as well as DNA vector. Eventually, the generation of knockout strains of *NR* or *APT* in this study is a technical proof of the feasibility of genome editing in this algal species.

## 2. Results

### 2.1. Generation of NR- or APT-Edited Mutants

The *NR* gene, encoding an enzyme that mediates the conversion of nitrate to nitrite, usually the rate-limiting step of nitrogen assimilation in higher plants and microalgae, was selected for the demonstration of a successful genome editing technology for *C. vulgaris* UTEX395. Additionally, *APT*, coding for a protein that catalyzes the conversion of adenine to adenylate, was also chosen as a target for genome editing because it is recognized as a promising marker gene. Targeting these genes in *C. vulgaris* UTEX395 facilitates the selection of mutant strains, as the disruption of *NR* or *APT* results in uninhibited growth in medium containing KClO_3_ or 2-fluoroadenine (2-FA), respectively [29,37].

**Figure 1:**
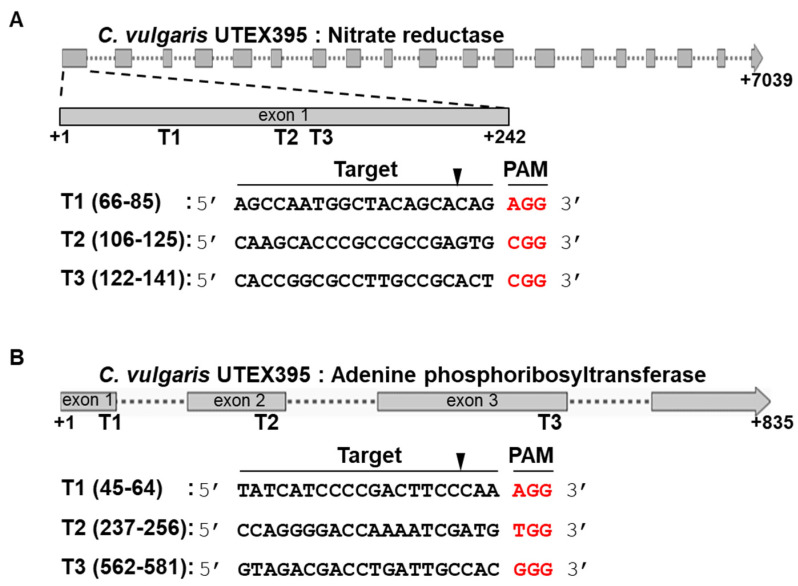
Design of the single guide RNA (sgRNA) sequences for genome editing of (**A**) *NR* and (**B**) *APT*. Twenty bp-long target sequences (black) and 3 bp-long PAM sequences (red) are presented below the illustration of the gene. Triangles indicate the cleavage sites.

**Figure 2:**
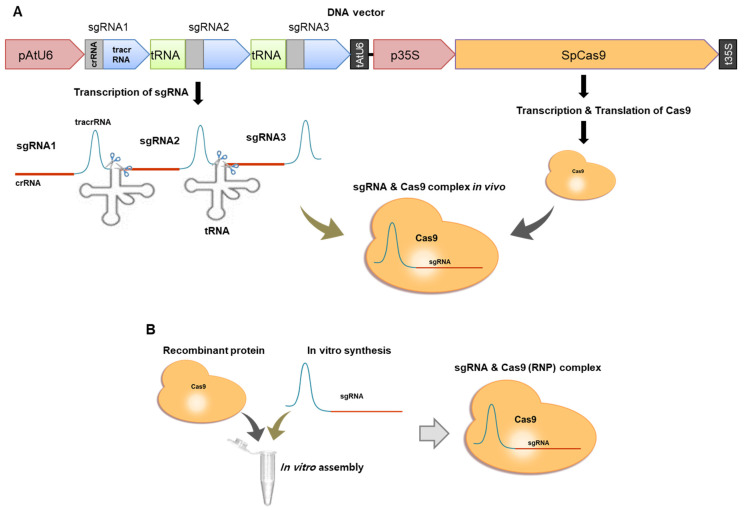
Schematic diagram of the preparation of ribonucleoprotein (RNP) complexes for genome editing. (**A**) Transcription and translation of sgRNAs and CRISPR associated protein 9 (Cas9) protein from the DNA vector. The sgRNAs and Cas9 protein will be assembled in vivo. (**B**) In vitro assembly of an RNP complex.

**Figure 3:**
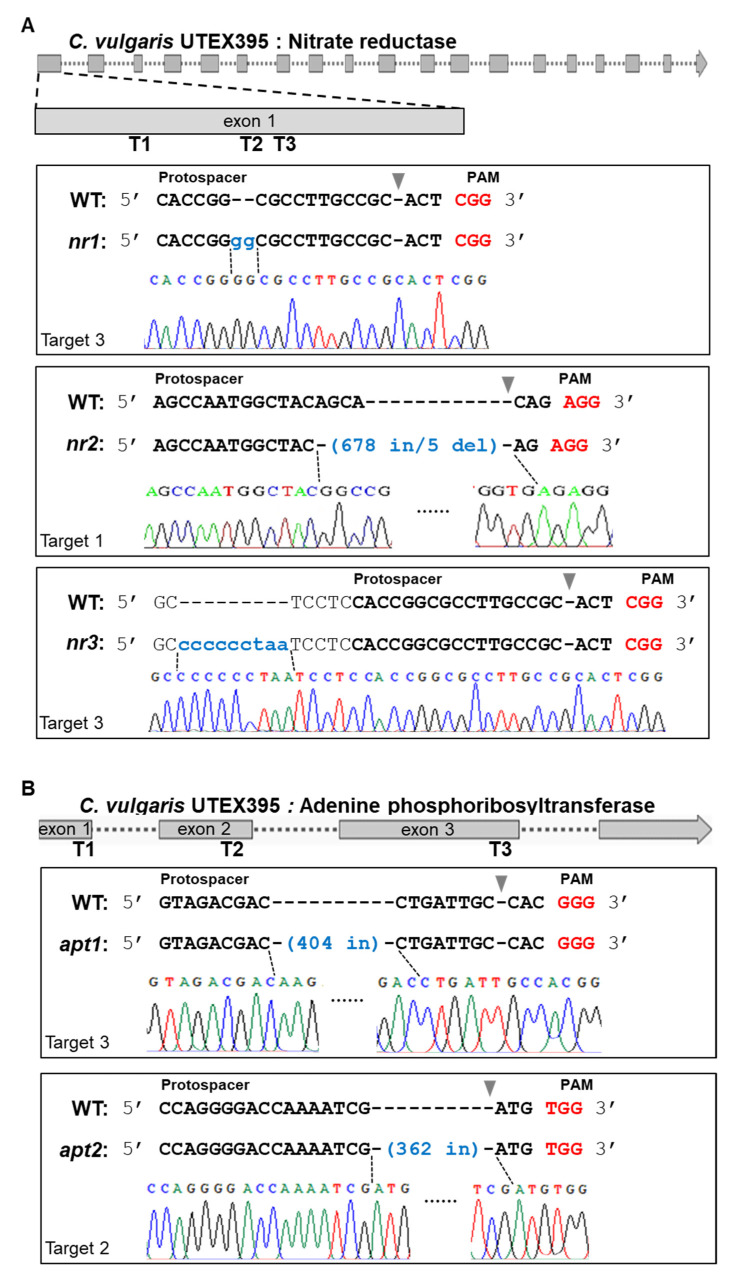
Sequence analysis of genome-edited mutants. (**A**) Edited genome sequences of nitrate reductase (*NR*) in *nr* mutants as determined by Sanger sequencing. The *nr1* and *nr3* mutants show a small insertion on target 3. The *nr2* mutant displays a 678 bp insertion and a 5 bp deletion at target 1. (**B**) Edited genome sequences of adenine phosphoribosyltransferase (*APT*) in *apt* mutants as determined by Sanger sequencing. The *apt1* mutant exhibits a 404 bp insertion at target 1, while *apt2* shows a 362 bp insertion at target 2. Reverse triangles indicate the RuvC cleavage sites.

NR-edited (*nr*) mutants were generated as described in the Materials and Methods Section using a DNA vector system or RNP complexes by electroporation. Three different sequences on exon1 were targeted (Figure 1A). *C. vulgaris* UTEX395 was transformed with either a circular Cas9-sgRNA expression vector or in vitro-assembled RNP complexes (Figure 2). Finally, three different mutants were selected by sequence analysis among hundreds of candidate colonies that resisted to KClO_3_ in several trials (Figure 3A). The *nr1* and *nr2* mutants were generated by the DNA vector system, and the *nr3* mutants were generated by the RNP complex system. *nr1* showed a 2 bp insertion in the protospacer region on target 3, while *nr2* showed a 678 bp insertion at the cleavage site with a 5 bp deletion on target 1. The inserted DNA belonged to the scaffold 853 of the whole genome sequence of *C. vulgaris* UTEX395. In *nr3*, a 9 bp insertion was discovered in the region of target 3. Because of these frameshift mutations, the translation of *NR* was terminated early on exon 1 or exon 3. Additionally, we tried protenolistic bombardment to the same target, however the *NR* edited mutant was not obtained. On the other hand, the *APT*-edited (*apt*) mutants were generated by proteolistic bombardment with RNP complex targeting three different sequences on exon 1–3 (Figure 1B). After performing three independent bombardment experiments, two different mutants were identified among an average of 32 ± 16 colonies obtained by screening on selective agar medium containing 2-FA. Analysis of the edited *APT* sequences (Figure 3B) highlighted a 404 bp insertion at the protospacer region on target 3 in *apt1*, whereas *apt2* showed a 363 bp insertion at the cleavage site on target 2. These inserted DNAs were reconstructed by the recombination of the cleaving DNA fragment at a target site, resulting in a repeated sequence of the *APT* gene. These mutations in *APT* resulted in the early stop or frameshift of amino acid translation. Notably, *nr1*, *nr3*, and *apt1* showed unexpected mutations placed outside the cleavage site, in accordance to previous reports of gene editing in microalgae [20,33,34] and higher plants [38,39]. Such mutations are thought to occur in the process of DNA repair by error-prone non-homologous end-joining after a double-strand DNA break [38].

### 2.2. Verification of Null Mutation of Genes in the Edited Mutants

**Figure 4:**
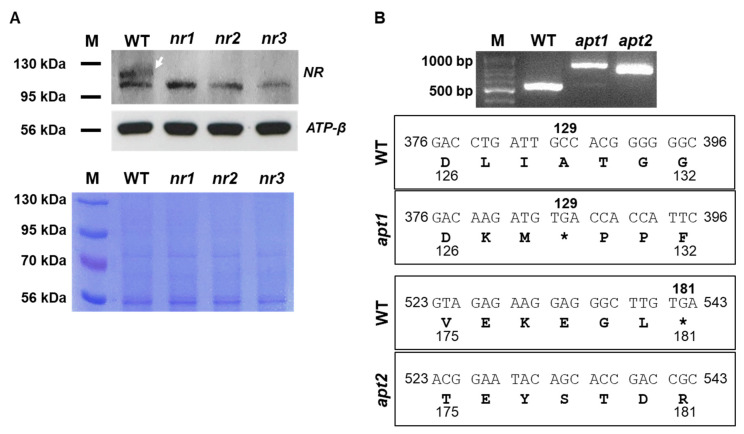
Expression of *NR* and *APT* in edited mutants compared with the WT. (**A**) Western blot analysis with a specific antibody against nitrate reductase (upper panel). Total protein content was assessed by Coomassie staining (lower panel). *ATP-β* was used as a reference protein. (**B**) Comparison of gene and amino acid sequence of *APT* between edited mutants and the WT. PCR amplification of *APT* from cDNA of *apt* mutants and WT cells (upper panel) and corresponding sequences (lower panels), showing the presence of frameshift mutations. M: size marker.

The protein expression of the edited genes was predicted to be abnormal due to mutations in the DNA sequence (Figure 3). Indeed, according to sequence analysis, the edited gene sequences led to prematurely terminated translation or to the generation of abnormal amino acids sequences. In the case of *nr* mutants, the termination codon would appear on exon 1 (*nr2*) or exon 3 (*nr1* and *nr3*). To verify the early termination of translation, Western blot analysis was conducted to compare NR protein levels between *nr* mutants and wild-type (WT) *C. vulgaris* UTEX395 (Figure 4A). We detected the expression of *NR* in WT. The antibody bound slightly above the 95 kDa size marker, corresponding to the predicted size of the native NR protein (~110 kDa). In contrast, *NR* expression was not detected in *nr* mutants. For the verification of gene expression in *APT*-edited mutants, the length and the sequence of *APT* mRNA was analyzed because there is no specific antibody to directly detect *APT* protein expression. The mRNA analysis corroborated the predictions of sequence analysis. An early termination codon was created on exon 3 in *apt1*, while the amino acid sequence was shifted abnormally after position 130 in *apt2* (Figure 4B and Supplementary Figure S4). Additionally, 74-aa peptides were added to the C terminus of the mutated protein in *apt2*. These results indicated that the target genes were fully knocked out by genome editing.

### 2.3. Physiological Analysis of NR- and APT-Edited Mutants

**Figure 5:**
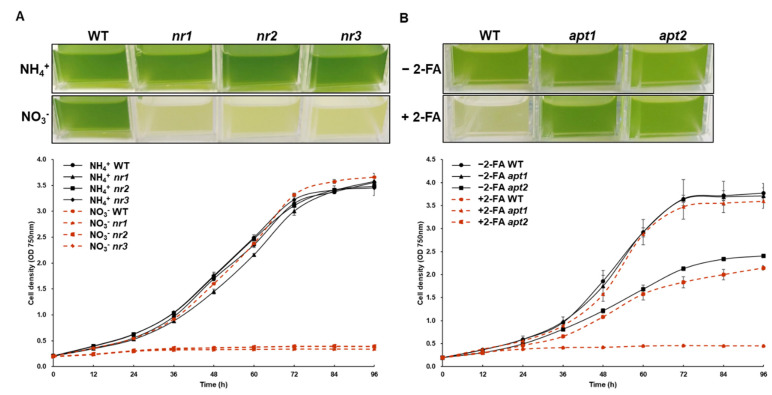
Growth analysis of NR- and APT-edited mutants compared to the WT. (**A**) The growth of nr mutants was fully inhibited in nitrate-supplemented medium. (**B**) Determination of cell growth of *apt* mutants in 2-fluoroadenine (2-FA) containing medium. *apt* mutants acquired resistance to 2-FA and could grow well, whereas the growth of WT was fully suppressed. These results indicated that the edited genes were not functional. Growth measurement was performed triplicate independently (*n* = 3).

As mentioned above, if the *NR* gene does not function, the cells cannot utilize nitrate, and thus their growth is inhibited in media supplemented with nitrate as the only nitrogen source. Therefore, to test changes in the physiological characteristics of *C. vulgaris* UTEX395 upon genome editing, the growth of WT cells and *nr* mutants in nitrate-supplemented medium was evaluated (Figure 5A). In the medium containing ammonium as the sole nitrogen source, no significant difference in growth between WT and mutant cells was observed. In contrast, the cell growth of *nr* mutants was completely inhibited in medium containing nitrate as the sole nitrogen source, while WT cells grew normally. This result clearly indicated that the *NR* gene was knocked out in the *nr* mutants, consistently with the absence of NR protein expression. The loss of *NR* gene function in *nr* mutants could be easily verified due to their auxotrophy. However, since *APT* is not an essential gene for survival, the loss of function of the *APT* gene in *apt* mutants could be verified only by reconfirming 2-FA resistance in liquid medium (Figure 5B). This assay confirmed that in liquid medium supplemented with 100 µm of 2-FA, as in the screening process, *apt* mutants showed normal growth, while WT cells grew more slowly.

The *nr* mutants generated in this study not only highlight the successful genome editing of *C. vulgaris*, but also represent a valuable resource for *C. vulgaris* genetics since the screening of transgenic strains employing the *NR* gene would allow to avoid the use of exogenous selection markers (e.g., antibiotic resistance genes) [40]. Therefore, these auxotrophic strains could be used as convenient platforms for the development of genetic modification tools based on superior selection criteria compared to antibiotics. These methods could be used to accelerate the development of transgenic lines with less concern about environmental pollution.

## 3. Materials and Methods

### 3.1. Microalgal Strain and Culture Conditions

*Chlorella vulgaris* UTEX395 (obtained from a culture collection of the University of Texas, Austin, USA) was grown mixotrophically in tris-acetate-phosphate (TAP) medium. WT and all genome-edited strains were cultivated in filter-capped flasks under continuous light (80–100 μmol photons m^−2^ s^−1^) at 25 °C with shaking at 100 rpm for maintenance. To validate NR functionality in *nr* mutants, cell growth was evaluated in a medium supplemented with 20 mM NH_4_^+^ or NO_3_^−^. To validate *APT* functionality in *apt* mutants, cell growth was compared in liquid media with or without 2-fluoroadenine (2-FA).

### 3.2. Identification of Target Gene Sequences and Design of sgRNAs

The *NR* sequence of *C. vulgaris* was found in the nucleotide database (Accession: U39930) of the National Center for Biotechnology Information (https://www.ncbi.nlm.nih.gov/). To identify the *NR* sequence of *C. vulgaris* UTEX395, the published *NR* sequence was aligned to the whole genome sequence of *C. vulgaris* UTEX395 (BioProject accession: PRJNA186385). Then, the *NR* sequence of *C. vulgaris* UTEX395 was determined by genomic DNA and cDNA sequence analysis (Supplementary Figure S1). Also, the *APT* sequence of *C. vulgaris* UTEX395 was newly identified in this study (Supplementary Figure S2). Whole exons of both genes were determined by comparison of gDNA and mRNA sequences.

The sgRNA sequences for the implementation of CRISPR-Cas9 were designed by Cas-Designer (http://www.rgenome.net/cas-designer/). Previous reports recommended choosing genome editing targets located in the early coding sequence (CDS) region to induce an earlier translational stop codon by frameshift mutation [30,31,32]. Based on this strategy, we determined three different targets for *NR* editing located within exon1 (Figure 1A), while the target sequences dispersed on three exons of *APT* were chosen owing to its short gDNA sequence (Figure 1B). To avoid off-target effects, nonspecific binding sequences were predicted using Cas-OFFinder (http://www.rgenome.net/cas-offinder/). According to Cas-OFFinder analysis (mismatch: 2 bp), all the sgRNAs applied in this study were not predicted to bind to off-target regions. Therefore, we excluded any off-target effects. 

### 3.3. Vector Construction for Implementation of the CRISPR-Cas9 System

Codon-optimized cas9 (Supplementary Figure S3) (synthesized in this study; Bioneer Inc., Daejeon, Korea) from *Streptococcus pyogenes* was cloned in a DNA vector using restriction enzymes and T4 DNA ligase using traditional cloning protocols (Figure 2A). The cas9 sequence was cloned downstream of the *Cauliflower mosaic virus* (CaMV) 35S promoter sequence in the empty vector at the *Xba* I and *Bam*H I restriction sites. This was cloned into *Spe* I and *Stu* I restriction sites to be located downstream of the *Arabidopsis thaliana* (At) U6 terminator. A tRNA-processing system, which is an intrinsic mechanism that forms small RNA precisely in living cells, was applied to transcribe multiple sgRNAs at the same time [41]. The pUC57-pre tRNA vector (kindly provided Professor Zanmin Hu, China) comprising the DNA fragment of the tracrRNA-fused tRNA was used as a template for producing sgRNA-tRNA DNA fragment by PCR. Next, the sgRNAs designed were cloned at downstream of the AtU6 promoter by Golden Gate cloning via digestion with a *Bsa* I restriction enzyme. The primer sequences used for sgRNA cloning are listed in Supplementary Table S1.

### 3.4. In Vitro Assembly of Ribonucleoprotein Complexes

For RNP complex delivery through electroporation, 100 μg of lyophilized Cas9 protein (ToolGen, Seoul, Korea) dissolved in nuclease-free water and 70 µg of in vitro-transcribed sgRNA (GeneArt™ Precision gRNA Synthesis Kit, Invitrogen, Carlsbad, CA, USA) were mixed and incubated at room temperature for 10 min to form an RNP complex [30]. The RNP complex was kept on ice before electroporation. 

For RNP complex delivery through proteolistic bombardment, we prepared RNP complexes of Cas9 protein (4 µg) and each pair of sgRNAs (2 + 2 µg) in 20 µL of Cas9 reaction buffer (20 mM HEPES, 100 mM NaCl, 5 mM MgCl_2_, 0.1 mM EDTA, pH 6.5) and incubated the mixture at 25 °C for 10 min. We performed an experiment to target two gene locations at the same time by constructing an RNP complex comprising two sgRNAs. Then, the RNP complexes were mixed with 20 µL (1.2 mg) of pre-washed gold microcarrier particles (0.6 µm of diameter, Bio-Rad, Hercules, CA, USA). The gold particles coated with RNPs were equally loaded onto four macrocarriers and dried on a clean bench.

### 3.5. Generation of Genome-Edited Mutants by Electroporation

Since selection using KClO_3_ requires the induction of *NR* expression, *C. vulgaris* UTEX395 was adapted for three days in BG11 medium containing 6 g L^−1^ glucose under continuous light at 25 °C with shaking at 100 rpm. Cells in the logarithmic phase (2.0–2.5 × 10^7^ cells mL^−1^) were collected by centrifugation at 2000× *g* for 5 min at room temperature (22 °C). The cell pellet (2 × 10^8^ cells) was washed with a hyperosmotic buffer (0.2 M sorbitol and 0.2 M mannitol) and incubated in 20 mL of cold hyperosmotic buffer for 1 h on ice. The pretreated cells were then harvested by centrifugation at 2000× *g* for 5 min at 4 °C. The cells were resuspended in 200 µL of electroporation buffer (0.2 M sorbitol, 0.2 M mannitol, 0.08 M KCl, 0.005 M CaCl_2_, and 0.01 M HEPES) [42]. To transform cells with DNA vectors or RNP complexes, 5 µg of DNA together with 25 µg of sperm DNA or pre-assembled RNP complex was mixed with 100 µL of cell suspension. The mixture was transferred to a 2 mm gapped electroporation cuvette and kept on ice for 10 min. Electroporation was carried out at 3.3 kV·cm^−1^, 25–50 μF, and 200 Ω using a Gene-Pulser apparatus (Bio-Rad, Hercules, CA, USA). After electroporation, the cell suspension was recovered by incubation in BG11 medium containing 6 g L^−1^ glucose in the dark overnight at 33 °C [20]. Recovered cells were spread on BG11 agar plates supplemented with 20 mM NaNO_2_ and 100–150 mM KClO_3_ to select the genome-edited mutants. After colonies formed, all colonies were transferred to liquid medium with or without NH_4_^+^ for the second screening.

### 3.6. Generation of Genome-Edited Mutants by Proteolistic Bombardment

Proteolistic bombardment of RNP complexes was performed according to our previous report [31], with slight modifications. In brief, *C. vulgaris* UTEX395 was cultivated in liquid medium under continuous light at 25 °C with shaking at 100 rpm. Cells for *NR*-targeting experiments were cultured in BG11, while cells for the *APT*-targeting experiment were cultured in TAP medium. Cells in the logarithmic phase (2.0–2.5 × 10^7^ cells mL^−1^) were collected by centrifugation at 2000× *g* for 5 min at room temperature. The cell pellet (1–2 × 10^7^ cells) was spotted on solid agar medium. After the coated gold particles had entirely dried on the macrocarrier, proteolistic bombardment was carried out using the Biolistic Particle Delivery System PDS-1000/He (Bio-Rad, Hercules, CA, USA) fitted with 1100–1350 psi rupture discs. After bombardment, cells were incubated for 20–24 h on solid agar medium and cultured by dilution on selective medium. The selective medium was as described above for the *NR*-targeting experiment, and TAP medium containing 100 µM 2-FA for the *APT*-targeting experiment. Cells that underwent bombardment grew on the solid medium for approximately 2–3 weeks. Colonies were collected and transferred onto fresh selective medium for the second screening.

### 3.7. Verification of Genome-Editing by DNA Sequence Analysis

To verify the mutations at the genomic DNA level, DNA sequencing was performed on cells that appeared to show the desired physiological characteristics at the second screening. Genomic DNA was extracted using the cetyl trimethylammonium bromide (CTAB) method, which is a common method for isolating DNA from plant tissues [30]. Targeted genes were amplified by PCR using specific primers for each gene. All primers used for the identification of the edited gene sequences are listed in Supplementary Table S2. The PCR product was purified by agarose gel electrophoresis and sent to Macrogen Inc. (Seoul, Korea) for Sanger sequencing.

### 3.8. Verification of Gene Knockout by Protein or RNA Levels

To verify efficient knockout of *NR*, Western blotting against NR protein was performed. Cultivated cells (1 × 10^6^ cells) were boiled in SDS-PAGE loading buffer, separated by electrophoresis on 8% SDS-polyacrylamide gels, and transferred to a PVDF membrane using a XCell II Blot Module (Thermo Fisher Scientific, Waltham, MA, USA). A nitrate reductase antibody (AS08-310, Agrisera, Vännäs, Sweden) was used to estimate protein levels. An antibody against the beta subunit of ATP synthase (*ATP-β*) (AS05-085, Agrisera, Vännäs, Sweden) was used as a reference. An HRP-conjugated goat anti-rabbit IgG (H+L) antibody (#31460, Thermo Fisher Scientific, Waltham, MA, USA) was used as secondary antibody. NR and ATP-β protein were visualized on an X-ray film by chemiluminescence using EPD (Enhanced Peroxidase Detection) Western Reagent (Elpis Biotech, Daejeon, Korea).

To verify the mutation of *APT*, the lengths of the correspondent mRNA and their sequence were analyzed. Cultivated cells were harvested by centrifugation at 2000× *g* for 5 min at room temperature. The cell pellet was immediately frozen in liquid nitrogen. RNA was extracted with a Hybrid-R kit with Trizol (GeneAll, Seoul, Korea) according to the manufacturer’s protocol. cDNA was synthesized by reverse transcription with a random hexamer (EBT-1511, Elpis Biotech, Daejeon, Korea). The primers used for the amplification of *APT* mRNA molecules by PCR are listed in Supplementary Table S2.

### 3.9. Statistic Analysis

To validate the technical error of the experiment, the growth experiment was carried out through 3 independent flask cultures.

## 4. Conclusions

Genome editing and effective DNA transformation systems in the reliable industrial species *C. vulgaris* expand the reverse genetics toolbox of industrial microalgae and can inspire numerous applications in synthetic biology based on carbon capture and conversion. In this study, we verified the applicability of genome editing techniques that had not yet been well established in *C. vulgaris* UTEX395 through the knockout of two different genes (*NR* and *APT*). Low transferring efficiency of a genetic molecule into the cell and ineffective expression of genes still act as high barriers to gene editing in this species. Therefore, it is still necessary to increase and optimize the efficacy of DNA or protein transfer systems for this algal species. The application of improved gene editing tools such as proteolistic method and improvement of promoter sequence and efficient gene expression cassette will increase the utilization of the system developed and verified in this study. Gene editing using CRISPR-Cas9 and an effective DNA vector system in the reliable industrial strain, *C. vulgaris*, expands the reverse genetics toolbox of industrial microalgae and can inspire numerous possibilities in systemic biology, synthetic biology, and carbon capture and conversion.

## Data Availability

Data is contained within the article and supplementary material.

## Supplementary Materials

Supplementary Materials can be found at https://www.mdpi.com/1422-0067/22/2/480/s1, Figure S1: Sequence of the nitrate reductase gene of *C. vulgaris* UTEX395. The *NR* sequence is 7039 bp long and contains 19 exons, indicated in yellow, Figure S2: Sequence of the adenine phosphoribosyltransferase gene of *C. vulgaris* UTEX395. The *APT* sequence is 835 bp long and contains four exons, indicated in yellow, Figure S3: Sequence of the codon-optimized *Streptococcus pyogenes cas9* synthesized in this study, Figure S4: Sequence analysis of the adenine phosphoribosyltransferase gene of *C. vulgaris* UTEX395, Table S1: Primer sequences for the construction of the *cas9* expression vector, Table S2: Primer sequences for Sanger sequencing of edited genes.
