# Supplementary material for: Establishment of a Genome Editing Tool Using CRISPR-Cas9 in Chlorella vulgaris UTEX395
Source: Int J Mol Sci. 2021 Jan 6;22(2):480. doi: 10.3390/ijms22020480 (PMC7825080; doi:10.3390/ijms22020480)
Supplement: Supplementary file 1 [file ijms-22-00480-s001.pdf]

Supporting information

Establishment of a Genome Editing Tool Using CRISPR-Cas9 in *Chlorella vulgaris* UTEX395

Jongrae Kim †, Kwang Suk Chang †, Sangmuk Lee and EonSeon Jin *

Department of Life Science, Research Institute for Natural Sciences, Hanyang University, Seoul 04763, Korea; kjr1210@hanmail.net (J.K.); kschang@hanyang.ac.kr (K.S.C.); warmmuk@naver.com (S.L.)

* Correspondence: esjin@hanyang.ac.kr; Tel.: +82-2-2220-2561

† These authors contributed equally to this work.

Figure S1

T1
atgacagtgctcctggcaggcgaggactcagcccacggcagcgggtctgcaggagcagcaatgaaagccaatggc
T2 T3
tacagcacagaggcaccagcagcatcgccgcaagcacccgccgccgagtgcggcaaggcgccggtggaggagccg
ctgcagcccggcactgacgagtacaagctgcacctgcccgtcaccgaagtgctggacgcagacaagggcaccaag
gacgagtggatcccccggtgagctctcatactgcggctcaagactgcgagccaggcggccggcggctgggctgcg
gctggctcccccgtgcacctctgctcatgcctttctgcatgggtgcagcagctctgccgcgctgtgtcggcccgg
agattgtgcatctttgcagcagatgcattcctcctctgcaccttcctggcccctcacctgccggcgctgcaaaca
cgtcgcacctccttgcatggctgcacagcctgtgtcgcctcttgccactcaacgcctcccccctgctgccctgccc
cctgcaggcacccagaccttgtgcggctcacagggcggcatcccttcaacgtcgagcccatcccgtcccgcctct
ttgagtcgtacatcacaccaccctccctgcactatgtacgcaaccacggagctgtaccgcagattaagtgggagg
agcaccgcctggcagtcaacggtgagggggcgtgtatggctagctcatagccaggtagcgtgtctagctggctag
ctatcgtgtccagctagcctgcaagtgtatatggctagcatggctgggcgggcagcctccacaacagcaggcagt
ggagccagccaggggtgggctttagggtttttttttgtaggggtttagggttctacagacaggaagggagcctc
cctctcttgcatccctcttgccgtgactgctgctcgctcggtgctctagccccgtcgttcagcgatgtctcacct
ttcgctgccctgtttgtttgcccttgttttcccgcaggcctggtttctgcgcccaccacattcacaatggacgac
atcctcgcgatgcctagtgttgacgtgacctgtacactgacctgcgcaggtgcagtggttggaggtggcttggcg
cgtgccaggctgcctgttgctgcctgttttgcctgccatcctgttttgttttggcagcaagtcacagactaacgc
tccagcattgccaaccacactggcccgtgcattgtttgttcaaaccggttgcctcctccctgctgtcagctgcgg
gctactgccggcacccgcttgcccactgagcactccctgcctttctccctgtactgcaggcaaccgccgcaagga
ggagaacatggtgaagcagacgataggcttcaactgggggccctcgggcacaggctgctccacctggacaggcgt
gcgcgtgagcgacttgctgcagcgctgcgggatgaagacgcccaaggagggcgcgcgccacgtgtgcttccgagg
cccaaagggtgcgtgctgccagctgccgaggcagaccttacaatgccgccagctgccgagacggagcctgcggtg
ctgcattgagtcagcgttcggcatgcgtcctctcccgccattgtgacttggcggccgcgcactgctcgcaattgt
ttcgtcgctcgtatcactcatgctgctgcatctcagcgtctcttccttgtcctttcttttcgttttgcaggcgag
ctgcccaagggagacgacggcagctacggcaccagcgtcacctgggtaaaggctatggacccagcctctgacgtc
atcctcgcatacaagcagaacggccgcctgctgacacccgaccacggctacccgctgcggctcatcattccaggc
tacatcggcgggcgcatgatcaagtggtgagcaggcctggctgtgctgtttaaaagcctggccacaatagcggga
gccgacggccatgcctctcctctctgctctggagacccccctcctgcccttgcccccctcctgcccttgctgtcat
tgttcctgcccttgcttgccttcactcattctctcactacttcctttcttatctttcttcttcctttgcaggctg
gaggagatcacggtcaccgaggtggagagccagaactactaccacttccatgacaacagagtgctgccctcgcac
gttgatgaggcgctggccaactccgaaggtgggcgcgcgcagggctgtgtgtgcgtgtatggttgtacggggttt
caggcgtgtgtaggggtaggtgcaggagttctggaggtgtgcgccaggttacagtgtgtgctggtgtggtgttgg
tgttgctggctggggcattgcagctacatcagtgggccgggcgaccaggtgggctgcaagcgtgtgctggtaaat
ggtggcggtgcgtggaggcgttgccggctcagtgtataactgcatcgtctgcacgtgcccttcgtttctttttgc
tggctcaaggtcagcctctgcccaccgcacctctggctgcaggctggtggtacaagccggacttcatcatcaatg
acctcaatgtccagtcagccataggctacccggcacatgaagaggtgttgcccctcgcggccggcacctacgccg
tacgaggctacgcgtactgcggtgagagccgcgtgtacagcacaccctgcgtacttactgcacaacgcccgcccg
ggtgccgcacaagcaccgactggcggttccagcctgcagtttgccaccccggccatgcgcttggcgctctcctcc
tccatgcttgcctctgcattccccccctgcgcgtcatttccctcccgccaaggaaacaagtctctgctccctttgc
aggcaacggcaacaagatcattcggtgcgaggtttcgctggacgacggcaagagctggcggctgggctctgtgac
gcacgaggggcaacctactgagtacggcaagcactggggctgggtgtggtggagcttggaggtgcccattggtga
gtgagagtgtaactagccacaggcgctgggctgatgttgttgcgccgtgcgtacgaacacaccgccgttgcacca
gtcgtgcgtgcgcagcagcacttcacaagcatcaagctggttgcgctgctttgctggaggctagcctgcttgctt
gcccctgcttgcctgcgcctcacctgggcgctgaggaccccgcctgcttgctgccgcgcctcccttccccacccg
gctgtgctgcagctgagctgcttaccactcctgaaatcatttgccgagcctgggacagctccatgaacacacagc
ccaacacgttcacctggtgggtgtgcctgcctccccacctccccctccatgcctgctgcctgttgcctcacctgc
tggcttcagctgcccgagcgcctcagcctgcgacactccgtgcggcaattggctgcctgcccggaccacttgcca
gccgcacggcactttgagcctgggtcgctgttcaccagtcacgccgcttctgttctgtttccacctgagccctcg
cttgcctgaagctcctcattcatttcatctctcacctttcctccacctcacctgtcctgcaggaacgtgatgggg
atgatgaacaactgctgctatcgcgtcaagatccaccccccgccagaccaccgacggccgctttgcgctgcagttt
gagcaccccaccattgccggccccactgtcggcggctggatgaaccgagcagaggacgtggcggcggcggcagca
gtgacggtaaggggggtccgggccgccctgtgctgctggacactgtgctgctcggcacatgaaccacacagcacag
gaggcatgtgtctgactaattgtgcagctgctgccgtgcgtcggttgtctggcaacgcaagcaaagcgcttttcc
tagttgttgcagcacgcctgcttcccgctcctgccctctgccgctgctgaaagtgctgctgccgcatgctttcac
caccatcaaccaaccgccaacctgatgctgctgaacttctttgttgctgcaggtggcgccgccacccgcgcccgc
aggtgccaagagcttcaccatggcagaagtggagcagcacacgaccatggagagcgcgtggtttgttgttgatgg
aaaggtgtacgacgcaacacccttcctgaaagaccacccgggtgagccattgctgtttttgttgacactgtcagt
gtgccccgcaaagtgacaggcgatacatacttggttatgcatccttgaccacctcctgctgctgcttcccccgcc

tcgcttctgcggtctcgtctgctttcgacccgcttccttgtattttccgtgctcactcggtgcaggtggcgccga
ctcgatcctgcttgtggctggcacagacgccactgacgagtttaatgccatccactcgctcaaagccaagaagca
gctgctggagtactacattggagagctggcggaagaggggcaggaggcagcagcaccagcgccggcgaccccggc
gccagcggcggcaatcggcacggcggtgccaggtgtgtgggcgcaatgctttggcccgggctgaactgagcacag
caggctgcgtcgcactcgctgccgcacacagcttgctgtggagctgcggtgtgcagcagtttcaagtgcagcgct
ggtcttggtcttaggacatccgccaccggtcttatgtcccacctccgccttgctagctcacgcgtgcgccactgc
tgccgcctcccgatcccatgtcgcagttgccaacggcgcagcgccagctgcagcagctccggaagagttggtggc
gctcacccccgcaagcggcagtcgttcaagctgattgagaaggaggcgctgtcacacaacacccgccgcttccg
attcgcactgcagtcgccgcagcaccgctttgggctgccggtcggaaagcacgtcttcctttatgccaagtgagc
gaggcagcagcagctgcggcagtgacacagcagcgccagcaggagtttgccctgccagaaggctggctggcgggc
tgctgtgtgggctgtgctgccggctgtgcaggacaccacagtgtagatggcgcgggttggcttgcatgggcagcg
ccctgttccgctgccctcccggctgcagccacttttcttgcgcttcctgccatgccaagcacccaccccaccctc
tgaccaccctttctcttgaccgccccccccttcatttgggcagggttgacggcgagctggtgatgcgggcttacac
cccctcatcctctgacgaccagctgggctactttgaactggtggtgaagatctacttcgcaaaccagcacccccg
tttcccagaaggaggtcagtcattcatgcctggctggctcgtgtggccgtggctgtggtcgtagctgcagctggc
ttgcttctgtggcgtgggcgtgagtgttgcggttgtgctcgtggtgccttggcaggctcggatttgaatgcatag
ctggtatcccacttacctgcacctattggcacgccagcgttccacctaccatcactccaccaacccatgtggctc
cccctgcctgtgtgtgcaggcaagatgagccagtaccttgagggaatggccatcggggactacatggaggtgaag
ggtccgctggggcacgttcactacactgggcgaggcaggtgagaagtagtttagggttgattgtgtgttggggtg
gggcctcgaaaggaggcagcagggatatgcagcaggaggggtgccatcatgcacgcagtggccttgcttgccttg
cctgcctgccctgcctgctgacctactgccccgcactccctcacagccctgcctcaacctttcttcctgccatgc
gtgccgctttgtgttgcagctacacgctggatggcacgccgcacagcgccagccgcatcagcatgattgctggcg
gcacaggcatcaccccccatgctccaggtgaacccttgaattgctcgctgctgacgccgctggtttgcaccgcgcc
atgtgcgccagcggcttcctccagggcttgcctgtggcccaggcccacgcacatgcaccacaatctccactgctg
cagctgccgcagcctgcttattgtgctcagttgtccctgttccatgtacccggcccctctgcccatcctgctgct
ttcctccttcctcctttctttcttcccgccaggtcattaaagcggtgctgaaggaccccaaagacacaaccgagc
tctccctgctctatgccaatgtgtcaccggatgacatcctgctgcgagaagagctggacgcactggcagccaagc
acgacaacttcagcgtgtggtacacaggtgcgctgtgctgcgacagtagcatcagctggcagctggtgggctggg
ccaggcggcaagggctggctggatgcagtgcgtgcagggttgctagatgggaggcagggagacttgggatgctgg
gacgtgcaagggactgtcagggaaaatcgctgtgcccctgcacaaacgcactgtggcgtgtggcgccgcttctct
ctgctctcgtccccttctcgccatgctcccacacccctgccttggtttgtggtgcagttgacaaggcggatgagg
ggtggccgttcagcaccgggttcatcaatgaggacatggtgaaggagcgcctgtttccgggtgagtacatattgt
ggtggcagcaaggcaccaccctgtgcggcagcagtgttgtgtgcagcggcggcctggatgtctgtgtctgcgttg
cgaataaacaaaaaaactgatttggtgaagacggttgggtgaagacatgtggtattggagggagacgagggctg
ctagtctggctgctcaatgtgcaagcttgctgctcctcttgtttgcttcttgacactcactcttcttctgccctg
cttgtccctgccctctcttgctgcagccggcgacgacaccatctgctgcctgtgcggccctcccccatgatcaa
gtttgcctgcctgcccaacctcgagaagctgggctacaagccggagcagtgcattcaattttga

Figure S1. Sequence of the nitrate reductase gene of *C. vulgaris* UTEX395. The *NR* sequence is 7,039 bp long and contains 19 exons, indicated in yellow. Target sequences for gene editing are highlighted by blue boxes.

Figure S2

T1
atgggggctgacccgcgcaccgcgctcatctcagacagcatccgtatcatccccgacttcccaaaggtgaggcgg
aaagtgatgatggtgcccgtcatgcattgtacatcgcagactcaacagcaatcagactcgcctcttcgtgtccag
gcagggatcatgtttcaagatgtgaccaccattcttctggatccggtcgccttcaaacacactgttgacatgctg

T2
catgagcggtaccaggggaccaaaatcgatgtggttgcaggtggggcgtgtggcaggcattgctaaaacagctta
tggcgcttagtcaaacctgcctactgaccaccaaccttccctggtacccctttttgtcacttgtgctgccgcagga
tttgaggcaagagggctcatatttggtgctcccctggccatcgcactggggtgcgcgtttgtgccactgcgcaag
cctgggaagctgccaggagacgtgctgtcagcagactacgtgacggaatacagcaccgaccgcatagagatgcat

T3
gtgggggcagtgcggcaaggacagcgagtgctgctggtagacgacctgattgccacgggggcacgctacgtgag
tgcctacagatatctcacacgggctgccatcacccaactggcagcacattgccagcatgtttgtctttgaatgac
catgccgttgcactgtgcaggagcgggtgtggagctggtgcagaaggcgggcgggcaggtagtcgaagcggcatg
cataattgagttgcccgagctcaagggcagggagaagctggagggcctgccactatttgtgctggtagagaagga
gggcttgtga

Figure S2. Sequence of the adenine phosphoribosyltransferase gene of *C. vulgaris* UTEX395. The *APT* sequence is 835 bp long and contains four exons, indicated in yellow. Target sequences for gene editing are highlighted by blue boxes.

Figure S3

atggactacaaggatcacgacggcgactacaaggaccacgacatcgactataaggacgacgacgacaagatggcc
cccaagaagaagcgaaaggtgggcatccacggggtgcccgctgccgacaagaagtacagcattggcctggacatc
ggaactaactctgttggctgggcagtgatcaccgacgagtacaaggtgccgtcgaagaagtttaaagtcctgggc
aacacagatcggcactccatcaagaagaacctcatcggcgcgctgctgttcgacagcggtgagacggccgaggca
acccggctgaagcgcaccgcccgcaggcgctacacccgccgcaagaaccgcatctgctacctccaggagatcttc
tccaacgagatggcgaaggtcgacgatagcttctttcaccgtctggaggagagcttcctggtggaggaggacaag
aagcacgagcgccatcccatcttcggcaacattgtggacgaggtcgcctaccacgagaagtacccaacgatctac
cacctgcggaagaagctggtggactccacagacaaggccgacctgcgcctgatctacctcgccctggcccacatg
atcaagttcaggggccactttctgatcgagggcgacctcaacccggacaatagcgacgtggacaagttgttcatc
cagctggtgcagacctacaaccagctcttcgaggagaaccccattaacgccagcggagtcgacgcaaaggcgatc
ctctccgctcgcctgtcgaagtctcgccgcctcgagaacctgatcgcacagctgccgggcgagaagaagaacggc
ctgttcgggaacctgatcgccctgagcctgggtctgacccccaacttcaagagcaactttgacctggcggaggac
gccaagctgcaactgtccaaggacacctacgacgacgacctggacaacctcctggcccagattggtgaccagtac
gctgacttgttccttgccgctaagaatctgagcgacgccatcctgctgtctgacattctgcgcgtgaacaccgag
attacgaaggcgcccctgagcgcatccatgatcaagcgctatgatgagcaccaccaggacctgaccctgctgaag
gcgctggtccgccagcagctccccgagaagtacaaggagatcttcttcgaccagtcgaagaacggctacgcaggc
tacatcgacggcggggcgagccaggaggagttctacaagtttatcaagccgatcctcgagaagatggacggcacg
gaggagctgctggtgaagctcaaccgcgaggatctcctccgcaagcagcggacatttgacaacggcagcatcccc
caccagattcacttgggggagctgcacgccatcctgcgccgccaggaggacttctacccgtttctcaaggacaac
cgcgagaagatcgagaagatcctgaccttccgcatcccttactacgtcggcccccctcgcgcggggcaactcccgc
ttcgcatggatgacccgcaagagcgaggaaaccatcacaccgtggaacttcgaggaggtggtggacaagggcgct
agcgcccagtcgtttatcgagcgcatgacgaacttcgacaagaacctgccaaatgagaaggtgctccccaagcac
agccttctgtatgagtactttacagtctacaacgagctgactaaggtgaagtacgtgaccgagggcatgaggaag
cccgcgttcctgagcggcgagcagaagaaggccatcgtggacctgctgttcaagaccaaccggaaggtcacggtt
aagcagctcaaggaagactacttcaagaagatcgagtgcttcgactcggtggagatctccggggtggaggaccgc
ttcaatgcctccctgggtacctaccacgacctgctgaagatcatcaaggacaaggacttcctcgacaacgaggag
aacgaggacatcctggaggacatcgtgctgaccttgactctgttcgaggatcgggagatgatcgaggagcgcctg
aagacctacgcccacctgttcgacgacaaggtcatgaagcagctgaagcgaaggcgctacaccggctggggccgc
ctgagccgtaagctgatcaacggcatccgggacaagcagagcggcaagacgatccttgacttcctgaagagcgat
ggctttgcgaaccgcaacttcatgcagctgattcacgacgacagcctcacatttaaggaggacatccagaaggca
caggtgagcggccagggcgacagcctgcacgagcacatcgcgaacctcgcaggctctcccgccatcaagaagggt
attctgcaaaccgtgaaggttgtggacgagctggtgaaggtcatgggccggcataagcccgagaacatcgtcatc
gagatggcccgggaaaaccagaccacccagaagggacagaagaacagccgcgagcgcatgaagcgaatcgaggag
ggcattaaggagctggggtcccagattctcaaggagcacccggtggagaacacgcagttgcagaacgagaagctc
tacctgtactaccttcagaacggccgcgatatgtatgtggaccaggagctggacatcaatcgcctgagcgactac
gacgtggatcacatcgtcccccagagcttcctgaaggacgattccatcgacaacaaggtcctgacacgctcggac
aagaaccgcggcaagtctgacaacgtgcccagcgaggaggtcgtcaagaagatgaagaactactggcgacagctg
ctgaacgcgaagctgatcacgcagcggaaattcgacaaccttaccaaggccgagcgcggcggcctctcggaactg
gacaaggctggcttcatcaagcgccagctggtcgagacacgccagatcaccaagcacgttgcgcagattctggat
agccggatgaacaccaagtacgacgagaacgataagctgattcgcgaggtgaaggtgatcaccctgaagtccaag
ttggtgtccgacttccgcaaggacttccagttttacaaggtgcgcgagatcaacaactatcaccacgcccacgac
gcctacctgaacgcagtggtgggcactgcgctgatcaagaagtacccgaagctcgagagcgagttcgtgtacggg
gactacaaggtgtacgacgtgcgcaagatgatcgcaaagtcggagcaggagattggcaaggctaccgcgaagtac
ttcttctacagcaacatcatgaacttcttcaagacagagatcaccctggccaacggcgagatccggaagcgcccc
ctgatcgagacaaacggcgagacgggagaaatcgtgtgggacaagggccgcgatttcgccaccgtccgcaaggtc
ctgagcatgccacaggtgaacatcgtgaagaagacggaggtgcagaccggcgggttttccaaggagagcatcctg
ccgaagaggaacagcgacaagctgattgcccggaagaaggactgggacccgaagaagtacggtgggtttgattcg
cccactgttgcgtactctgtcctggtggtggcaaaggtcgagaagggcaagtccaagaagctcaagtccgtgaaa
gagctgctggggatcaccattatggagcgcagctctttcgagaagaatccgatcgactttctggaggcgaaaggc
tataaggaggtgaagaaggacctgatcattaagctccccaagtactccctcttcgagctggagaacggccgcaag
cgcatgctggcctccgcaggcgagcttcagaagggcaacgagctggccctgccgtccaagtacgtgaacttcctc

tacctggctagccattacgagaagctcaagggcagccccgaggacaacgagcagaagcagctgttcgtcgagcag
cacaagcactacctggacgagatcatcgagcagatttcggagtttagcaagcgcgtgatcctcgcggacgccaac
ctggataaggtgttgagcgcatacaacaagcaccgcgacaagccaatccgggagcaggcggagaacatcatccac
ctcttcaccctgacgaacctgggcgccccggccgcctttaagtacttcgacaccaccatcgaccgcaagcgctac
accagcacaaaggaggtgctggacgccaccctgatccaccagtccatcaccggcctgtacgagacgcgcattgac
ctgagccagctgggggggcgataagcggcccgcggccaccaagaaggcgggccaggccaagaagaagaagtga

Figure S3. Sequence of the codon-optimized *Streptococcus pyogenes cas9* synthesized in this study.

Figure S4

A

```
  1 atg ggg gct gac ccg cgc acc gcg ctc atc tca gac agc atc cgt atc atc ccc gac ttc 60
  1  M   G   A   D   P   R   T   A   L   I   S   D   S   I   R   I   I   P   D   F  20

 61 cca aag gca ggg atc atg ttt caa gat gtg acc acc att ctt ctg gat ccg gtc gcc ttc 120
 21  P   K   A   G   I   M   F   Q   D   V   T   T   I   L   L   D   P   V   A   F  40

121 aaa cac act gtt gac atg ctg cat gag cgg tac cag ggg acc aaa atc gat gtg gtt gca 180
 41  K   H   T   V   D   M   L   H   E   R   Y   Q   G   T   K   I   D   V   V   A  60

181 gga ttt gag gca aga ggg ctc ata ttt ggt gct ccc ctg gcc atc gca ctg ggg tgc gcg 240
 61  G   F   E   A   R   G   L   I   F   G   A   P   L   A   I   A   L   G   C   A  80

241 ttt gtg cca ctg cgc aag cct ggg aag ctg cca gga gac gtg ctg tca gca gac tac gtg 300
 81  F   V   P   L   R   K   P   G   K   L   P   G   D   V   L   S   A   D   Y   V 100

301 acg gaa tac agc acc gac cgc ata gag atg cat gtg ggg gca gtg cgg caa gga cag cga 360
101  T   E   Y   S   T   D   R   I   E   M   H   V   G   A   V   R   Q   G   Q   R 120

361 gtg ctg ctg gta gac gac ctg att gcc acg ggg ggc acg cta cga gcg ggt gtg gag ctg 420
121  V   L   L   V   D   D   L   I   A   T   G   G   T   L   R   A   G   V   E   L 140

421 gtg cag aag gcg ggc ggg cag gta gtc gaa gcg gca tgc ata att gag ttg ccc gag ctc 480
141  V   Q   K   A   G   G   Q   V   V   E   A   A   C   I   I   E   L   P   E   L 160

481 aag ggc agg gag aag ctg gag ggc ctg cca cta ttt gtg ctg gta gag aag gag ggc ttg 540
161  K   G   R   E   K   L   E   G   L   P   L   F   V   L   V   E   K   E   G   L 180

541 tga
     *
```

B

```
  1 atg ggg gct gac ccg cgc acc gcg ctc atc tca gac agc atc cgt atc atc ccc gac ttc 60
  1  M   G   A   D   P   R   T   A   L   I   S   D   S   I   R   I   I   P   D   F  20

 61 cca aag gca ggg atc atg ttt caa gat gtg acc acc att ctt ctg gat ccg gtc gcc ttc 120
 21  P   K   A   G   I   M   F   Q   D   V   T   T   I   L   L   D   P   V   A   F  40

121 aaa cac act gtt gac atg ctg cat gag cgg tac cag ggg acc aaa atc gat gtg gtt gca 180
 41  K   H   T   V   D   M   L   H   E   R   Y   Q   G   T   K   I   D   V   V   A  60

181 gga ttt gag gca aga ggg ctc ata ttt ggt gct ccc ctg gcc atc gca ctg ggg tgc gcg 240
 61  G   F   E   A   R   G   L   I   F   G   A   P   L   A   I   A   L   G   C   A  80

241 ttt gtg cca ctg cgc aag cct ggg aag ctg cca gga gac gtg ctg tca gca gac tac gtg 300
 81  F   V   P   L   R   K   P   G   K   L   P   G   D   V   L   S   A   D   Y   V 100

301 acg gaa tac agc acc gac cgc ata gag atg cat gtg ggg gca gtg cgg caa gga cag cga 360
101  T   E   Y   S   T   D   R   I   E   M   H   V   G   A   V   R   Q   G   Q   R 120

361 gtg ctg ctg gta gac gac aag atg tga cca cca ttc ttc tgg atc cgg tcg cct tca aac 420
121  V   L   L   V   D   D   K   M   *                                             128
```

C

```
  1 atg ggg gct gac ccg cgc acc gcg ctc atc tca gac agc atc cgt atc atc ccc gac ttc 60
  1  M   G   A   D   P   R   T   A   L   I   S   D   S   I   R   I   I   P   D   F  20

 61 cca aag gca ggg atc atg ttt caa gat gtg acc acc att ctt ctg gat ccg gtc gcc ttc 120
 21  P   K   A   G   I   M   F   Q   D   V   T   T   I   L   L   D   P   V   A   F  40

121 aaa cac act gtt gac atg ctg cat gag cgg tac cag ggg acc aaa atc gat gtg gtt gca 180
 41  K   H   T   V   D   M   L   H   E   R   Y   Q   G   T   K   I   D   V   V   A  60

181 gga ttt gag gca aga ggg ctc ata ttt ggt gct ccc ctg gcc atc gca ctg ggg tgc gcg 240
 61  G   F   E   A   R   G   L   I   F   G   A   P   L   A   I   A   L   G   C   A  80

241 ttt gtg cca ctg cgc aag cct ggg aag ctg cca gga gac gtg ctg tca gca gac tac gtg 300
 81  F   V   P   L   R   K   P   G   K   L   P   G   D   V   L   S   A   D   Y   V 100

301 acg gaa tac agc acc gac cgc ata gag atg cat gtg ggg gca gtg cgg caa gga cag cga 360
101  T   E   Y   S   T   D   R   I   E   M   H   V   G   A   V   R   Q   G   Q   R 120

361 gtg ctg ctg gta gac gac ctg att gcc acg ggg ggc acg cta cga ttt gag gca aga ggg 420
121  V   L   L   V   D   D   L   I   A   T   G   G   T   L   R   F   E   A   R   G 140

421 ctc ata ttt ggt gct ccc ctg gcc atc gca ctg ggg tgc gcg ttt gtg cca ctg cgc aag 480
141  L   I   F   G   A   P   L   A   I   A   L   G   C   A   F   V   P   L   R   K 160

481 cct ggg aag ctg cca gga gac gtg ctg tca gca gac tac gtg acg gaa tac agc acc gac 540
161  P   G   K   L   P   G   D   V   L   S   A   D   Y   V   T   E   Y   S   T   D 180

541 cgc ata gag atg cat gtg ggg gca gtg cgg caa gga cag cga gtg ctg ctg gta gac gac 600
181  R   I   E   M   H   V   G   A   V   R   Q   G   Q   R   V   L   L   V   D   D 200
```

```
601 ctg att gcc acg ggg ggc acg cta cga gcg ggt gtg gag ctg gtg cag aag gcg ggc ggg 660
201 L   I   A   T   G   G   T   L   R   A   G   V   E   L   V   Q   K   A   G   G  220

661 cag gta gtc gaa gcg gca tgc ata att gag ttg ccc gag ctc aag ggc agg gag aag ctg 720
221 Q   V   V   E   A   A   C   I   I   E   L   P   E   L   K   G   R   E   K   L  240

721 gag ggc ctg cca cta ttt gtg ctg gta gag aag gag ggc ttg tga                     765
241 E   G   L   P   L   F   V   L   V   E   K   E   G   L   *                       254
```

Figure S4. Sequence analysis of the adenine phosphoribosyltransferase gene of *C. vulgaris* UTEX395. (A) Gene and protein sequences of wild-type *APT*. (B) Edited mRNA sequence of *APT* gene in *apt1*. (C) Edited mRNA sequence of *APT* gene in *apt2*. Edited sequences do not show the expression of right amino acid of *APT*. Red alphabet indicates the stop codon.

Table S1. Primer sequences for the construction of the *cas9* expression vector.

Name	Sequence (5′ → 3′)
sgRNA NR-**F1**	ATATATGGTCTCGattgAGCCAATGGCTACAGCACAGGTTTTAGAGCTAGAAATAGC
sgRNA NR-**R1**	ATTATTGGTCTCGCACTCGGCGGCGGGTGCTTGTGCACCAGCCGGGAATC
sgRNA NR-**F2**	ATATATGGTCTCGagtgGTTTTAGAGCTAGAAATAGC
sgRNA NR-**R2**	ATTATTGGTCTCG**aaac**AGTGCGGCAAGGCGCCGGTGTGCACCAGCCGGGAATC

* Gray box: sgRNA sequence
* Red box: tRNA sequence
* Green box: tracrRNA sequence
* Red characters: GGTCTC, *Bsa* I recognition sequence

Table S2. Primer sequences for Sanger sequencing of edited genes.

Name	Sequence (5′ → 3′)
CvNR-F	CAACTCCTCCCTCATACTCCTCGCAC
CvNR-R	CCACTCGTCCTTGGTGCCCTTGTC
CvAPT-F	CCGTCCACATCCAGTCAACA
CvAPT-R	TGAGCTCGGGCAACTCAATT
